# Exploring the promise and reality of ward-based primary healthcare outreach teams conducting TB household contact tracing in three districts of South Africa

**DOI:** 10.1371/journal.pone.0256033

**Published:** 2021-08-13

**Authors:** Candice M. Chetty-Makkan, Daniel deSanto, Richard Lessells, Salome Charalambous, Kavindhran Velen, Sewele Makgopa, Dumile Gumede, Katherine Fielding, Alison D. Grant

**Affiliations:** 1 The Aurum Institute, Johannesburg, South Africa; 2 School of Public Health, Faculty of Health Sciences, University of the Witwatersrand, Johannesburg, South Africa; 3 Health Economics and Epidemiology Research Office (HE^2^RO), Johannesburg, South Africa; 4 Africa Health Research Institute, KwaZulu-Natal, South Africa; 5 TB Centre, London School of Hygiene & Tropical Medicine, London, United Kingdom; 6 KwaZulu-Natal Research Innovation & Sequencing Platform, School of Laboratory Medicine and Medical Sciences, University of KwaZulu-Natal, Durban, South Africa; 7 Durban University of Technology, Centre for General Education, Durban, South Africa; 8 School of Laboratory Medicine and Medical Sciences, College of Health Sciences, University of KwaZulu-Natal, Durban, South Africa; York University, CANADA

## Abstract

**Background:**

Tuberculosis (TB) household contact tracing is a form of targeted active case-finding for which community health workers (‘outreach teams’) in South Africa are primarily responsible for its implementation. We conducted an exploratory qualitative study to understand the role of outreach teams in delivering TB household contact tracing.

**Methods:**

The study took place in three districts of South Africa between May 2016 and February 2017. We conducted 78 in-depth interviews (IDI) (comprising 35 key stakeholders, 31 TB index patients and 12 HHCs) and five focus group discussions (FGD) (40 outreach team members in four FGDs and 12 community stakeholders in one FGD).

**Results:**

Outreach teams contributed positively by working across health-related programmes, providing home-based care and assisting with tracing of persons lost to TB care. However, outreach teams had a limited focus on TB household contact tracing activities, likely due to the broad scope of their work and insufficient programmatic support. Outreach teams often confused TB household contact tracing activities with finding persons lost to TB care. The community also had some reservations on the role of outreach teams conducting TB household contact tracing activities.

**Conclusions:**

Creating awareness among outreach workers and clinic personnel about the importance of and activities related to TB household contact tracing would be required to strengthen the delivery of TB household contact tracing through the community-based primary health care teams. We need better monitoring and evaluation systems, stronger integration within a realistic scope of work, adequate training on TB household contact tracing and TB infection prevention control measures. Involving the community and educating them on the role of outreach teams could improve acceptance of future activities. These timely results and lessons learned should inform contact tracing approaches in the context of COVID-19.

## Introduction

Tuberculosis (TB) remains a global public health concern. In 2018, ten million individuals fell ill with TB. South Africa, specifically was identified as one of eight countries that contributed two-thirds of new TB cases worldwide [1, 2]. Scaling up active case-finding efforts are thus urgently required if the End TB strategy targets are to be realized [1]. Contacts of TB patients have an increased risk of developing TB compared to the general population and have consequently been identified for targeted active case-finding [3]. TB household contact tracing, a form of targeted active case-finding has been shown to detect and prevent TB cases [3, 4].

In South Africa, TB household contact tracing is implemented by Ward-based Primary Healthcare Outreach Teams (WBPHCOTs), commonly referred to as ‘outreach teams’. Employing outreach teams form part of a larger strategy introduced in 2011 to re-engineer primary health care [5], with the aim of improving the delivery of preventative health services to communities [6]. Outreach teams comprise of a professional nurse (usually assigned the role of outreach team leader) who supervises a group of health promotion practitioners and community health workers. Each outreach team is linked to a health facility and is responsible for extending health services to approximately 1,500 households through regular visits [7, 8]. The outreach teams receive general training [9] and are assigned to provide health services in the area where they reside. Contact tracing is among the many tasks that outreach teams are expected to complete and the extent of these contact tracing activities varies by province. The outreach teams get a wide scope of training which includes many other activities beyond contact investigation–this is part of the problem; and no refresher training provided. Some of the roles assigned to the outreach teams include health promotion, community awareness and education [10], screening and support for general illnesses including HIV and TB [11], treatment of minor illnesses, family planning [11, 12] and basic first aid for emergencies [5]. One success of the WBPHCOT programme is that marginalised communities and those living in remote or rural areas receive public health services [8]. Despite national efforts to provide public health services to the community, general challenges with transport, resources, accountability and supervision of outreach teams are evident [8]. There is limited information on the role of outreach teams for delivering TB household contact tracing activities and community perceptions to the services they provide. We conducted a study to understand the role of the outreach teams for conducting TB household contact tracing in South Africa.

## Methods

We conducted an exploratory qualitative study between May 2016 and February 2017 to understand delivery of TB household contact tracing and, specifically, to what extent the outreach teams are involved in TB household contact tracing in Ekurhuleni (Gauteng), Bojanala (North-West) and uMkhanyakude (KwaZulu-Natal) districts. We will use the term ‘outreach teams’ to broadly refer to individuals that provide outreach activities across the study districts such as community caregivers, community health workers, ward-based outreach teams (WBOTs) and Ward-based Primary Healthcare Outreach Teams (WBPHCOTs). None of the selected districts were a National Health Insurance (NHI) pilot district. The NHI is a proposed system that will provide all South African citizens with essential health care regardless of the individual’s ability to pay for the health services.

We set out to get information from a wide range of health care providers and health care users in the three districts. We purposively selected key and community stakeholders and invited them to take part in the study via telephone. Key stakeholders included staff actively involved in managing TB programme activities such as TB household contact tracing at provincial, district, sub-district and facility level. Community stakeholders were selected in one district only, and were defined as individuals who were perceived to represent the community in which TB household contact tracing was being implemented. Facility staff from the different public health clinics identified the outreach teams and arranged the focus group discussions (FGDs), at a neutral location that was convenient for all outreach teams to attend. Since all stakeholders were part of the health system or community, the interviewer and note-taker had previous contact with them.

We also identified TB index patients and their household contacts from clinic records and research staff set up appointments to interview them. The TB patients were purposively selected at the healthcare facilities (TB unit) when they visited the facilities for observation. The TB nurses gave the TB patients basic information about the study and if interested they were referred to the study research assistants. Thereafter the patients were given detailed information about the study and invited to participate. TB patients were purposively selected to include patients with different types of TB and gender groups. Selection continued until saturation was reached. Prior to the study, research staff had no contact with the TB index patients or household contacts. In-depth interviews (IDIs) with TB index patients or their household contacts took place at their homes or at venues that they preferred.

Face-to-face IDIs and FGDs conducted by authors (SM (Basic degree), CMC (PhD), KV (PhD), DG (Masters) and RL (MD)) and the research team took place in the language chosen by the interviewee (English, isiZulu, isiXhosa or Setswana), administered once and not repeated. The interviewers included three co-investigators, two co-ordinators and fourteen research assistants; that comprised of both males and females. The co-investigators had a PhD or medical degree and co-ordinators had either a basic or Master’s degree. The research assistants had approximately two years’ experience in qualitative data collection. Interview and focus group discussion guides were developed by reviewing literature and using components of the Consolidated Framework for Implementation Research (CFIR). Digital recordings were transcribed and when needed translated to English. After the interviews and FGDs, a discussion took place with the interviewers regarding their interaction with the participants. Interviews and FGDs lasted about an hour. TB index and household contacts did not provide feedback on the completed transcripts. However, stakeholders, mainly Department of Health staff and outreach workers provided feedback and/or clarity on the preliminary themes at a stakeholder meeting. Authors (CMC, DDS, SC) assessed saturation of themes during data collection and analysis. Those who participated in the FGDs were reimbursed ZAR100 (approximately $10) to compensate them for their time and travel. Outreach teams and community stakeholders received refreshments during the FGDs.

### Patient and public involvement statement

Participants were involved with the reporting and dissemination plans of our research.

### Ethics approval and consent to participate

University of the Witwatersrand Human Research Ethics Committee (ref. HREC 160305), the Biomedical Research Ethics Committee of the University of KwaZulu-Natal (ref. BE246/16) and the London School of Hygiene & Tropical Medicine (ref. 11020) approved the study. Department of Health in KwaZulu-Natal (HRKM 353/16), Gauteng (GP-2016RP1_403) and North West Province (NW_2016RP59_358) provided district approvals. All participants provided written informed consent prior to study participation.

### Data collection

All participants signed an informed consent form to participate and gave permission for digital recording of the session. Interview (S1 File) and FGD (S2 File) guides were used to explore the existing TB services provided at community level; motivators and barriers to TB diagnosis and treatment of household contacts; internal or external factors that influenced TB household contact tracing; gaps in TB household contact tracing systems and suggestions or plans for improvement of TB services. For TB index patients and household contacts, we further explored knowledge and experience of TB (including TB contacting tracing), knowledge and experience of HIV (including HIV counselling and testing and preference for HIV counselling) and preferences for any additional health services during contact tracing visits.

### Data analysis

English was the primary language used for the analysis. During the analysis, review of digital recordings and process notes took place (CMC and DDS). QSR NVIVO 10 software was used. Themes were developed by using inductive and deductive approaches to thematic analysis. For reliability, two authors (CMC and DDS) coded the original transcripts. We used an iterative approach to develop the codebook and index the transcripts according to themes and patterns which emerged. We retained codes of mutual agreement between the coders, while removing those that lacked inter-rater reliability. Independent reviewers who were not part of the original coding checked the codes. Revision of the codebook took place before finalising the themes. We show a summary of the themes and direct quotes from the participants to support the findings. Participant attributes that were further analysed included differences across districts and gender.

## Results

Table 1 summarises the total number of enrolled participants from Ekurhuleni, Bojanala and uMkhanyakude districts. We conducted IDIs with 35 key stakeholders (directors, managers, TB co-ordinators, facility-based staff), 31 TB index patients and 12 household contacts. We interviewed 18 (58%) male and 13 (42%) female TB index patients with mean age of 36 years old (range 19–63 years old). There was an equal distribution of gender for the household contacts. We conducted five FGDs where four FGDs were conducted with outreach teams only and one with community stakeholders. The one FGD with community stakeholders took place in the uMkhanyakude district. Out of the 40 outreach team members who took part in four FGDs, 39 were female outreach team members, one was a male outreach team member. None of the participants dropped out of the interviews or FGDs.

**Table 1:** Total number of enrolled participants from Ekurhuleni, Bojanala and uMkhanyakude districts.

Participants	Type of data collection	Ekurhuleni	Bojanala	UMkhanyakude	Total
Department of Health Stakeholders	Interviews	5	4	5	14
Facility Staff	Interviews	10	5	6	21
TB Index Patients	Interviews	9	10	12	31
Household contacts	Interviews	3	4	5	12
Outreach teams	Focus group discussion (FGD)	1	1	2	4
Outreach teams	Number of participants per FGD	10	12	18	40
Community stakeholders	Focus group discussion (FGD)	0	0	1	1
Community stakeholders	Number of participants per FGD			12	12

The following dominant themes developed. Although outreach teams contributed positively to the public health system, there was limited focus on activities specific to TB household contact tracing and poor community acceptance of their role. Reflected below is a description of the themes and supporting sub-themes with direct quotations.

### Main Theme 1: Positive contribution of outreach teams to public health service delivery

Outreach teams reported enjoying working with the community and made personal sacrifices to assist people in need. Residents also indicated that they valued the home-based care that they received.

“*In fact*, *we are the ones that brought people to the clinic so that the clinic will be full*. *We can make them cough and we won’t refuse because we love the community*. *What we are striving for is that they live* (recover).*”* FGD, Outreach team member, uMkhanyakude.

“*What I heard was that they said that there is no more pension for sick people that is why I take food from my household and carry for my patient when going to check him/her*. *I do this so that at least he/she can get one meal a day for him/her to take pills but fortunately enough he finally recovered and he is now alive in the community*.” FGD, Outreach team member, uMkhanyakude.

*“The elderly people in our community love us* (referring to the outreach teams*) so much*. *There is this other grandmother who had TB; whenever she sees me she makes sure that she greets me even if I do not see her and she would get happy to see me because she is now healed*.*”* FGD, Outreach team member, Bojanala.

*“They (referring to the outreach teams) encouraged*, *us if they are ladies they are like our sisters or your aunt*. *They like your family*, *the way they talk to you*. *They even empathize with you they feel the pain when you sick and act like your mother*. *They don’t have time to make a joke about the situation*, *you can even see their facial expression that what they say comes from the bottom of their heart*, *that’s why I’m saying they study their job very well*. *Yeah*, *they are important and they encourage us a lot*. *My father in law is having a certain disease … they said its cancer so these people (referring to the outreach teams) they visit him to clean and bath him*. *They also give him food to eat it and things that he might need*. *They also check if there is a progress in his life so you can that they very helpful*.*”* Interview, TB Index. Ekurhuleni.

“*They are well accepted as I have explained before that households are not the same*, *there are households where you find that there is a person who very sick*, *you find that he/she (referring to the outreach teams) stays with kids…”* Interview, HHCT, uMkhanyakude.

In most cases, community members associated the primary role of outreach teams with providing medication, following up on those who are lost to follow-up during treatment and providing referrals or physically helping those who were impoverished or unable to care for themselves.

*“I think a CCG (*referring to the outreach team*) is a person maybe*, *who is able to help people who are unable to help themselves”* Interview, Household Contact, uMkhanyakude.

Key stakeholders confirmed that the outreach teams assisted with tracing persons lost to TB care. Yet, specific reference on activities related to TB household contact tracing was vague.

“*TB sister will give a list to the team leader or the TB Sister herself can actually write on the communication book all the patients that she wants to be traced*. *The community health care workers* (referring to outreach teams) *will then go to that book and take all those patients that are there go and trace them*, *and write feedback on that communication book so that the TB sister can see what happened to those patients that were traced*.” Interview, Department of Health key stakeholder, Ekurhuleni.

### Main Theme 2: Limited focus by outreach teams on TB household contact tracing

Reported duties of outreach teams ranged from physical care of the incapacitated, TB screening, HIV testing, screening for non-communicable diseases, delivering medication, referral for antenatal care, assisting facility staff with clerical duties, assisting with applications for social support and the need to conduct multiple home visits. Tracing of people lost to TB care was one of the multiple roles that outreach teams were expected to complete. Due to a broad scope of work, unrealistic expectations on work load, and lack of both human resources and materials, outreach workers did not seem to prioritise TB household contact tracing. Specific, direct mention of TB household contact tracing was also rare.

“…*with the community health workers* (referring to outreach teams) *they say it’s too much work for them because they’ve got other things to do*. *They are expected to report their household registration and whatever*, *whatever every month and then with the TB tracing [it] is another thing*.” Interview, Department of Health key stakeholder, Bojanala.

Other existing barriers such as a lack of transport, restricted number of outreach team members and health safety concerns on TB infection and prevention control (IPC) perpetuated the challenges that they experienced.

“*We don’t have full coverage actually…as I’ve just mentioned and if you look at our population*, *we don’t have enough community health workers or enough WBOTS* (referring to outreach teams)”. Interview, Department of Health key stakeholder, Ekurhuleni.

“*That is the first challenge*, *transport is a major problem that makes us not to visit that household after 6 months*. *Our biggest challenge is the transport*.” Interview, Facility staff, uMkhanyakude.

“*Yes*, *because the biggest problem* (in) *our area is* (that it is) *too scattered so we will not be able to cover all unless we can find enough resources …you find that we are visiting very few compared to the people that have TB*. *It doesn’t reach everyone*.” Interview, Department of Health key stakeholder, uMkhanyakude.

“*You find that CCGs* (referring to outreach teams) *would go to that person without protection all that is a big mistake* (for the) *government to fail to provide gloves*, *masks and all equipment to protect CCGs*.” FGD, Community stakeholder, uMkhanyakude.

Contributing factors to limited focus on TB household contact tracing were gaps in monitoring and evaluation, specifically related to documentation of TB household contact tracing indicators and supervision of outreach teams.

“…*I am not really sure as to what I would be seeing in the communication book*. *Is it the true picture of what is really happening*? *Are they really going to these addresses*? *And maybe its wrong addresses we haven’t yet gone deeply to go verify their outcome*. *So eish (*local expression) *I will say it’s not really clear that’s whether they are effective in TB tracing you know*. *…monitoring is really a huge challenge*, *it is a really a huge challenge… we don’t have* (a) *clear monitoring system in place yet*.” Interview, Department of Health key stakeholder, Ekurhuleni.

“…*And you know*, *that kind of feedback loop that monitoring system is still not yet working*.” Interview, Department of Health key stakeholder, uMkhanyakude.

“…*The monitoring and evaluation of TB* [needs] *to be improved*, *you must have a tool that is consistent*.” Interview, Facility Staff, Ekurhuleni.

“*So sometimes the community or the household members they get furious to say somebody was here asking the same question and now you are here asking the very same question or looking the very same things that the other person was looking for*.*”* Interview, Department of Health key stakeholder, Bojanala.

“…*There must be a system which will force the CCGs to report to the facility about contacts…and supervision*. *Because maybe some of the things*, *it’s poor supervision*.” Interview, Department of Health key stakeholder, uMkhanyakude.

Although outreach teams confirmed that they received training on TB symptoms and prevention of TB transmission, they lacked knowledge on specific aspects of TB household contact tracing and new developments in the field of TB. Most outreach team member’s felt that their role was to trace people lost to TB care.

*“We need to get a proper training on TB*. *Maybe it will reduce the rate of “defaulters because if we are properly trained on TB* [and] *when one defaults*, *I will be trained on how to trace and how to take care of him/her* (person lost to TB care) *and this will reduce TB defaulters*.*”* FGD, Outreach team member, Bojanala

“*So*, *what is lacking here in tracing people with TB*, *the health workers have little information in matters relating to TB management*. *Because you find that other workers do not know the symptoms…I am concerned about health workers they do not have sufficient knowledge about TB because if they had sufficient knowledge about TB we would not have such a problem about TB because a person would be teaching about TB in the ward (referring to an area region) all the time*.*”* Interview, Department of Health key stakeholder, uMkhanyakude.

### Main Theme 3: Community reservations concerning the role of outreach workers

Some TB index patients and their household contacts were not comfortable that outreach teams visiting them knew their community due to perceived stigma.

“People; they still have that thing of stigma. Like when they see us coming wearing our uniform a person will ask “Did my neighbour saw [see] you?” Maybe we come to check a kid but because there’s still that thing (stigma); people who are wearing uniform are looking for people who have HIV… Her mother ended up telling (pleaded) [to] me that I must no longer come [back] because when I go in her house, people are telling themselves that there’s a sick person [in that house].” FGD, Outreach team member, uMkhanyakude.

“Others have that attitude that I am sick and once I cough badly they say I will infect them with TB. Well I am not sure whether they say it jokingly but the way they treat me is not right… Yes, I am scared that it will be disclosed without me knowing and [I am] not ready.” Interview, TB index patient, Bojanala.

TB index and their household contacts questioned the method used to select outreach teams, felt that outreach teams were not professionals and did not want someone that they knew visiting them. Concerns about breaching confidentiality and lack of privacy frequently emerged.

“*And another thing that they face; that they work within the community where they are known*. *And some of the people do not accept them because they think they are there for gossiping; not to actually assist them*.” Interview, Department of Health key stakeholder, uMkhanyakude.

“*In the place [that I] am living in {XX} (name of section) … I don’t prefer the community care givers [be]cause they can know about my status and my disease… maybe I had a fight with someone who is in [the] community*, *[when the] care giver… comes to visit me …*, *she [finds] that I have TB or low blood [or] any disease…then she or he [can] go to the to someone else [and] tell that person about my status or my disease yah*” Interview, TB index patient, Bojanala.

TB index patients and household contacts did not value the role of outreach teams possibly due to feelings of resentment, as the outreach teams were selected for employment in communities where unemployment rates are very high. This may account for them not accepting the role of outreach teams in the community.

“*… they (referring to the outreach teams) said*, *we were abused in one of the household[s]*, *… she* (referring to person in the community) *chased them out like a dog*” Interview, Household contact, uMkhanyakude.

“*I become worried when they* (outreach teams) *go to the field*. *They come back and* (they are) *telling me that “In that house*, *they chased us with dogs”*. *I* (then) *become worried*, *I don’t want to lie*. *The other one will say “I was so scared to even go in because* (it) *is looking so dangerous*. *Sometimes you* (are) *not sure whether you will be* (able) *to come out or you won’t come out”*, *things like that make me* (get) *so worried*. *I don’t want to lie about these things; and they do report them*.” Interview, Facility staff, Ekurhuleni.

“*They (referring to the outreach team) will not be accepted*, *because we as people do not see things the same way*, *and we do not like same things*. *Maybe let’s say I am a community care giver if I come to check people for TB*, *there will be someone who will refuse… When I arrive maybe the one who will refuse*, *he/she will send dogs … there is a person who don’t like to be checked in front of his/her family*, *he/she would like to hide it… [if I] tell them (referring to the outreach team) things that are confidential and instruct them not to tell anybody about those things*. *Should that person (referring to the outreach team) leak that thing because you may find that it won’t take even a month before he/she leaks it*, *then he will be told that no you don’t qualify to work here*, *you (referring to the outreach team) will kill people*…” Interview, TB index patient, uMkhanyakude.

“…Y*ou* (referring to outreach teams) *want to be paid because of us* (patient) *… because he/she* (patient) *has that mentality that as we* (community caregiver) *are working in the community… how we were hired*? *People think if you want to help them you will get paid because of them*, *they have that attitude*”. FGD, Outreach team member, uMkhanyakude.

“*They* (the patients) *get angry when we arrive at their houses and they would chase us away*. *Shout at us and tell us to get out of their yards because they came to the clinic yesterday”* FGD, Outreach team member, Bojanala.

“*My suggestion so that life can be easy* (is that) *there must be days where team leaders go with us to the field*. *Until people get used to us that we are working at the clinic…You even feel so inferior as if you don’t know your work*. *So I feel like the professionals can meet us half way in our work*.” FGD, Outreach team member, Ekurhuleni.

## Discussion

We identified multiple roles of outreach teams and therefore gaps in their delivery of TB household contact tracing in South Africa. Due to a broad scope of work, unrealistic expectations, lack of both human resources and materials, outreach teams did not prioritise TB household contact tracing. Outreach teams did however provide home-based general care and assisted with tracing of persons lost to TB care. TB household contact tracing activities specifically, were often confused with tracing of persons lost to TB care. Contributing factors that limited the priority of TB household contact tracing were gaps in monitoring and evaluation, specifically related to documentation of TB household contact tracing indicators and outreach team supervision. Outreach teams also acknowledged having limited knowledge on specific aspects of TB household contact tracing. There was poor community acceptance of outreach teams and concerns around a lack of patient privacy. Creating awareness on the importance of identifying contacts of infected individuals through TB household contact tracing are urgently needed for informing future systematic approaches to contact tracing.

While we acknowledge that challenges with the outreach team approach are known, our study identified challenges specific to TB household contact tracing within different communities. Our findings concur with previous reports of insufficient human resources, infrastructure and material provision within the outreach teams system structure [8, 13, 14]. Since outreach teams provide multiple services to the community [10–12], there could be unrealistic expectations of their role, especially considering the relatively limited training of this cadre of staff. Our study confirmed that the high workload and unrealistic expectations of outreach teams limited their understanding and implementation of TB household contact tracing activities. These are in keeping with findings from another TB household contact tracing study conducted in South Africa, where integrating HIV with TB services in households compromised the focus of TB case finding [15]. Although integration of health services are intended to ease and increase access for individuals, it should not be implemented at the expense of diluting health service delivery.

Our study identified reasons for poor community acceptance of outreach teams where these findings could inform the development of future patient centred approaches to contact tracing. According to the policy guidelines, outreach teams had to be placed in an area that was familiar to them [8]. Yet, there was limited information given to the community on the role of outreach teams. Most people from the community in our study associated the role of outreach teams with historical experience of home-based carers. Our findings highlight the need for stronger public/community engagement to improve understanding of outreach teams and community-based health services. TB index patients and their household contacts preferred home visits from someone who they did not know to ensure confidentiality. They expressed reservations toward the outreach teams as they were not consulted about who should visit their homes for TB household contact tracing. In a recent study, we found that TB household contact tracing was well accepted when provided by research staff who were not from the community [16]. In light of the new COVID-19 pandemic, policy makers should make sure that there are comprehensive, broad community engagement when setting up contact tracing programmes as this could encourage community acceptance and uptake of screening activities that may take place at the homes of individuals.

Although studies report a high risk of TB infection and disease among health care workers in South Africa [17], outreach teams have insufficient training on TB infection prevention and control measures, limited resources for the maintenance of hygiene and short supply of personal protective equipment [18]. Essential training for outreach teams could include a standardised program with specific emphasis on TB household contact tracing, physical safety when conducting tracing activities such as being provided with personal protective equipment [19] current updates in the field of TB [9, 20–22] and field-based training on TB household contact tracing [23]. An approach that includes directed, structured training on contact tracing activities would also be beneficial to the ongoing COVID-19 response.

Similar to other studies [20, 22, 24, 25] our findings also showed gaps in monitoring and evaluation of TB indicators, specific to TB household contact tracing. Lack of clinical stationery to document TB household contact tracing indicators, no consistent reporting structure and uncertainty of which TB indicators to report for contact tracing highlighted the challenges experienced by outreach teams. Although the Department of Health has forms and tick lists to monitor general services provided and management of outreach teams [8, 13, 26], there is no system to check the accuracy of the information completed. Most of the current clinical stationery is paper-based [26], time-consuming to complete [13], covers general public service delivery and has minimal information on TB household contact tracing. We observed a lack of formal and consistent supervision of outreach teams, consistent with previous studies [7, 14, 20, 23, 27–29]. Professional nurses need to juggle supervising outreach teams and carrying out clinical duties at their facility [8]. This will be particularly challenging in areas with substantial staffing shortages. Due to the perceived high workload of facility staff, they may not always have time to manage outreach teams [7]. Pursuing electronic data collection [13, 26] may have wider benefits to the health system, especially in the era of COVID-19 where contact tracing will become more important for outreach teams. Better integration of public health services and guidance to outreach teams could improve overall contact tracing activities within our communities.

## Strengths and limitations

Our study had several strengths. Firstly, previous studies have focussed on the general role of outreach teams delivering TB or HIV services broadly within sub-districts [6, 12, 21–25, 27, 28, 30–32], while we focussed specifically on their delivery of TB household contact tracing. With TB case finding being a high priority in South Africa and policies advocating for outreach teams to implement TB household contact tracing, our study provides important insights into their current limitations, including their acceptance in the community. Secondly, we were able to interview a large number of key and community stakeholders, TB patients and their household contacts from three districts in South Africa. This ensured that we explored and identified a wide variety of perspectives and themes required to understand TB household contact tracing and the roles of outreach teams. In addition, we were able to interview more TB index patients that were male compared to females, even though males are generally a hard to reach group for public health studies. Thirdly, we ensured data saturation which gave us confidence that we achieved representativeness in themes that emerged from interviews.

Our study had a few limitations. Firstly, outreach teams focussed their interview responses on their challenges in an attempt to have the research team assist with their employment situation. Despite this, their responses were consistent with other studies and reflected underlying variations in the implementation of WBPHCOT policy. Secondly, we interviewed majority female outreach teams and had a limited perspective on the experience of male outreach teams and while this may be an imbalance in our respondents, it does reflect the gender distribution of outreach team members, most of whom are women. We addressed these limitations by conducting a high number of interviews in order to guard against misleading responses.

## Conclusions

We found multiple obstacles to effective TB household contact tracing by outreach teams including unclear prioritisation within a very broad remit; poor coordination with other elements of the TB programme; lack of training and resources; lack of monitoring and evaluation; and suboptimal acceptability to community members. If outreach teams are to effectively support TB household contact tracing activities, there needs to be greater prioritisation and investment into the programme or another approach needs to be identified. Findings from this and other studies should inform the development of systematic contact tracing approaches, especially in the current context of COVID-19, where efficient contact tracing is a key part of reducing the incidence of all diseases that are transferred directly from one person to another.

## Supporting information

S1 File In-depth interview guides. (DOC)

S2 File Focus group discussion guide. (DOC)

S3 File COREQ checklist. (DOCX)

## List of abbreviations

TB: Tuberculosis
WBPHCOT: Ward-based primary healthcare outreach teams
IDI: In-depth interviews
FGD: Focus group discussion
PHC: Primary Healthcare
HIV: Human immunodeficiency virus
HHC: Household contact
DoH: Department of Health
CCG: Community caregiver
IPC: Infection prevention and contro
