# Supplementary material for: Exploring the promise and reality of ward-based primary healthcare outreach teams conducting TB household contact tracing in three districts of South Africa
Source: PLoS One. 2021 Aug 13;16(8):e0256033. doi: 10.1371/journal.pone.0256033 (PMC8362963; doi:10.1371/journal.pone.0256033)
Supplement: S1 File — (DOC) [file pone.0256033.s001.doc]

### S1 File

### INTERVIEW GUIDE: DEPARTMENT OF HEALTH STAKEHOLDERS

## OPTIMIZING THE EFFICIENCY OF HOUSEHOLD CONTACT TRACING FOR TB CONTROL IN SOUTH AFRICA

### Part I: Structured Interview

#### Introduction

Staff to say out loud before starting interview

- Thank you for agreeing to take part in our research
- We are interested in knowing about TB contact tracing. There are no right or wrong answers.
- You do not have to answer any questions if you don’t want to. Just say pass and I will move to the next question.
- Your answers to these questions may help us improve TB services.
- All of your answers will be kept confidential. Confidential means we will not tell anyone your answers outside the research group.

**Date of interview**:

**Interviewer**:

#### Time started (HHMM):

#### Questions

1. What is the title of your current position?
2. How long have you held this position?
3. Is providing TB services part of your mandate? If no, who is responsible for providing this?
4. How are TB services delivered at your level? (i.e. District, Provincial, National)
5. Is TB contact tracing considered an important TB intervention at your level? Explain.
6. What resources are available for TB contact tracing? How are these resources distributed across your level? Do you receive resources for such activities from external sources i.e. non-governmental?
7. Who is responsible for TB contact tracing at your level? What cadre of staff perform this activity?
8. How are TB contact tracing activities reported at your level? Are there statistics which are generated and reviewed? What frequency is this reported at? Is there any feedback of performance to staff in the programme? Does review of the statistics ever lead to specific action being taken?
9. Is TB contact tracing done on all newly diagnosed TB patients? Are certain patient groups prioritized? Is the smear status of a patient important in determining whether they are contact traced? Explain.
10. Is HCT being offered during at households who are being contact traced? Who is it offered to? Does this depend on the cadre of health care worker conducting the contact tracing?
11. Do you believe that TB contact tracing is reaching everyone requiring a household visit? What proportion of index TB patients would you estimate are being reached?
12. Do you believe that TB patients diagnosed among the households are successfully started on treatment? What proportion do you believe are started on treatment within 2 days of being diagnosed?

### Time ended (HHMM):

### Part II: In-Depth Interview

#### Introduction and Ground Rules

1. Obtain written informed consent first, before any data are collected.
2. Interviewer to introduce self. Thank you for taking the time to meet with us today. Our names are [*insert names*] __________ and we would like to talk to you about TB contact tracing. We are doing this project to understand how we might improve TB contact tracing in the community, and particularly to see whether there are better ways to deliver contact tracing. We are interested in your views regardless of whether you have or have not got experience of TB contact tracing. We want you to be as open and honest when answering. There are no right or wrong answers in this discussion. Please feel free to tell us what you think.
   - Interviewer to explain the ground rules and terms of confidentiality for the interview:
   - The participant does not have to answer any question they do not want to.
   - The information you share will be handled in confidence. (in secret)
   - When we report back on the information collected in this discussion, your comments will not be able to be linked to you specifically.
   - We ask that you also agree not to share anything discussed in this room with others.
3. The discussion should take around one hour.
4. Interviewer to inform the interviewee that the in-depth interview will be tape recorded to make sure that all themes are captured. Turn the tape on and ask for verbal permission again to tape record, while the tape is running to verbally capture consent (this is a double check against the written consent). We will be recording the session because we don’t want to miss any of your comments. Although one of us may take some notes while we talk, we can’t write fast enough to get everything down on paper. As we are recording, please try to speak loudly so that we don’t miss your comments.

#### Themes to be explored

1. Motivators and barriers to diagnosis and treatment of household contacts
2. Internal or external factors that influence HHCT
3. Gaps in HHCT
4. Suggestions and plans for improvement/change

#### Time started (HHMM):

#### Questions

- 1. **Motivators and barriers to diagnosis and treatment of household contacts**
     - *Do you believe that TB contact tracing is being delivered optimally? If yes or no, explain.*
     - *Can you tell us who the cadre of staff are that are delivering TB contact tracing services?*
     - *What is your opinion of the cadre of staff who are conducting these activities?*
     - *What is the role of community care givers (CCG’s) in performing any TB contact tracing? Can you describe any change in role or future role of CCGs delivering contact tracing services?*
     - *How would you feel about integrating the CCGs and WBOTs with the TB programme to deliver contact tracing? Do you know of anywhere that has been done? What do you think would be challenges or factors of success by integrating these roles?*
     - *Would you have any concerns or positive views about CCGs being more involved with TB contact tracing?*
     - *Can you describe the training and resources that staff receive to perform TB contact tracing? In your opinion, are there sufficient resources and adequate training available to perform TB contact tracing? How do you think availability of resources and training could be changed to be more effective? Explain.*
     - *TB contact tracing can be time consuming; are staff accepting of delivering such activities? Elaborate.*
     - *Can you describe the benefits and challenges of TB contact tracing and should we continue with this? How can tracing of TB in the community be improved?*
     - *What role do the local clinics play in supporting TB contact tracing activities?*
  2. **Internal or external factors that influence household contact tracing**
     - *How are sputum specimens transported from the households to the National Health Laboratory Services (NHLS)?*
     - *Are sputum specimens ever discarded by the NHLS? How often does this happen and for what reasons?*
     - *Are TB results ever misplaced or lost? What happens in such an instance?*
     - *Are there other organizations that assist in delivering TB contact tracing activities? Please describe the assistance that they provide*
     - *If there are other organizations that support TB contact tracing, how is their data collected and included in your level of reporting?*
  3. **Gaps in household contact tracing and suggestions for improvement**
     - *Can you describe any gaps which exist in the way contact tracing is being delivered currently*
     - *Describe the current integration of HIV services with TB contact tracing. Describe your opinion of the current system of integration by elaborating on the positive and negative aspects?*
     - *Can you suggest any missed opportunities for offering other services while conducting household visits for TB contact tracing?*
     - *Can you describe to us what systems are in place that monitors the TB contact tracing activities? In your opinion what are the challenges and positive factors of these systems (M&E, etc.) in monitoring and/or improving TB contact tracing activities? Explain.*

***Any other comments***

Are there any final thoughts you have about TB household contact tracing?

***End of session***

Now we have come to the end of our discussion. Thank you for your participation. If you have any questions about your study participation, please contact us. Thank you.

#### Time ended (HHMM):

### S2

### INTERVIEW GUIDE: HEALTH CARE WORKERS

## OPTIMIZING THE EFFICIENCY OF HOUSEHOLD CONTACT TRACING FOR TB CONTROL IN SOUTH AFRICA

### Part I: Structured questionnaire

#### Introduction

Staff to say out loud before starting interview:

- Thank you for agreeing to take part in our research
- We are interested in knowing about TB contact tracing. There are no right or wrong answers. You do not have to answer any questions if you don’t want to. Just say pass and I will move to the next question.
- Your answers to these questions may help us improve TB services.
- All of your answers will be kept confidential. Confidential means we will not tell your answers to anyone outside the research group.

**Date of interview**:

**Interviewer**:

#### Time started (HHMM):

#### Questions

1. What is your job title?
2. How long have you worked at this facility?
3. Are you currently working in TB? If so, how long have you worked in TB?
4. How is TB contact tracing delivered at present at this facility?
5. Who is involved in TB contact tracing?
6. Are certain groups of people prioritized for TB contact tracing, e.g. drug-resistant cases or smear-positive cases?
7. Is HIV testing incorporated into TB contact tracing activities at the moment?
8. Who is responsible for coordinating TB contact tracing activities?
9. How are data/statistics collected on TB contact tracing?
10. Who are these data/statistics reported to and how frequently?
11. What proportion of households of people with TB would you estimate receive contact tracing at the moment?

#### Time ended (HHMM):

### Part II: In-depth interview

#### Introduction and Ground Rules

1. Obtain written informed consent first, before any data are collected.
2. Interviewer to introduce themselves. Thank you for taking the time to meet with us today. Our names are [*insert names*] and we would like to talk to you about TB contact tracing. We are doing this project to understand how we might improve TB contact tracing in the community, and particularly to see whether there are better ways to deliver contact tracing. We are interested in your views regardless of your experience of TB contact tracing. We want you to be as open and honest when answering. There are no right or wrong answers in this discussion. Please feel free to tell us what you think.
3. Interviewer to explain the ground rules and terms of confidentiality for the interview:
   1. The participant does not have to answer any question they do not want to.
   2. The information you share will be handled in confidence.
   3. When we report back on the information collected in this discussion, your comments will not be able to be linked to you specifically.
   4. We ask that you also agree not to share anything discussed in this room with others.
4. The discussion should take around one hour.
5. Interviewer to inform the interviewee that the in-depth interview will be tape recorded to make sure that all themes are captured. Turn the tape on and ask for verbal permission again to tape record, while the tape is running to verbally capture consent (this is a double check against the written consent). We will be recording the session because we don’t want to miss any of your comments. Although one of us may take some notes while we talk, we can’t write fast enough to get everything down on paper. As we are recording, please try to speak loudly so that we don’t miss your comments.

#### Themes to be explored

1. Perception of CCGs delivering HHCT services
2. Challenges of delivering HHCT services
3. Description of their understanding of how HHCT services should reach the community
4. Motivators to HHCT services being provided

#### Time started (HHMM):

#### Questions

1. Can you identify the main challenges to conducting TB household contact tracing?

- *Are there any suggestions that you have on how these challenges could be addressed?*

1. How do you think delivery of TB contact tracing could be improved?
   - *In your opinion, what would be the best way for contact tracing to be delivered?*
2. What is your understanding of the role of community care givers (CCGs)?
   - *Can you tell me more about any experience you have of working with CCGs?*
3. Have you had any experience of CCGs being involved in TB contact tracing or TB screening in households?
   - *Can you tell me a bit more about your experience?*
4. What do you think of the idea of CCGs being more involved in TB contact tracing or TB screening in households?
   - *Can you explain this a bit more?*
   - *What could be the main benefits of this?*
   - *Would you have specific concerns?*
5. Can you describe the skills and knowledge that CCGs have that places them in an adequate position to deliver TB contact tracing services?
6. How would you describe the relationship between CCGs and clinic nurses?
7. Can you describe the support that CCGs receive from clinic nurses, and by their supervisors?
   - *Would you have any concerns about the role of CCGs in delivering TB contact tracing services?* (*Probe on confidentiality)*
8. Who do you think should be responsible for coordinating TB contact tracing activities?
   - *If CCGs were involved who should coordinate this?*
9. Describe your opinion of integrating HIV testing with TB household contact tracing?
   - *What are the benefits of this?*
   - *What might be the challenges to doing this?*
10. If HIV testing were included, how do you feel about CCGs being involved with this?
11. Can you think how the monitoring & evaluation of TB contact tracing could be improved?
12. If you could change just one thing about how TB contact tracing is done, what would it be and why?

***Any other comments***

Are there any final thoughts you have about TB household contact tracing?

***End of session***

Now we have come to the end of our discussion. Thank you for your participation. If you have any questions about your study participation, please contact us. Thank you.

#### Time ended (HHMM):

### S3

### INTERVIEW GUIDE: PEOPLE WITH TB

## OPTIMIZING THE EFFICIENCY OF HOUSEHOLD CONTACT TRACING FOR TB CONTROL IN SOUTH AFRICA

#### Introduction and Ground Rules

1. Obtain written informed consent first, before any data is collected.
2. Interviewer to introduce themselves: Thank you for taking the time to meet with us today. Our names are [*insert names*] _________), and we would like to talk to you about TB contact tracing. We are doing this project to understand how we might improve TB contact tracing in the community, and particularly to see whether there are better ways to deliver contact tracing. We are interested in your views regardless of your experience with TB contact tracing. We want you to be open when answering. There are no right or wrong answers in this discussion. Please feel free to tell us what you think.
3. Interviewer to explain the ground rules and terms of confidentiality for the interview:

- The Participant does not have to answer any question they do not want to.
- The information you share will be handled in confidence (in secret).
- When we report back on the information collected in this discussion, your comments will not be able to be linked to you specifically.
- We ask that you also agree not to share anything discussed in this room with others.

1. The discussion should take around one hour.
2. Interviewer to inform the interviewee that the in-depth interview will be tape recorded to make sure that all themes are captured. Turn the tape on and ask for verbal permission again to tape record, while the tape is running to verbally capture consent (this is a double check against the written consent). We will be recording the session because we don’t want to miss any of your comments. Although one of us may take some notes while we talk, we can’t write fast enough to get everything down on paper. As we are recording, please try to speak loudly so that we don’t miss your comments.

#### Themes to be explored

1. Knowledge and experience of TB (including TB contacting tracing and acceptability)
2. Knowledge and experience of HIV (including HIV counselling and testing and preference for HIV counselling)
3. Preference for any additional health services during a contact tracing visit

#### Time started (HHMM):

#### Questions

1. Can you tell me what you understand about how TB spreads from person to person?
2. When you were diagnosed with TB, were you worried about the health of your household members?
   - *How important do you feel it is for your family to be checked for TB?*
   - *Could you explain your answer?*
3. Could you describe whether anything happened for your household members after you were diagnosed with TB?
   - *Did they receive a visit at home to check them for TB?*
   - *Or were they advised to visit a clinic or hospital to be checked for TB?*
4. If there are children at home, can you describe whether anything happened specifically for the children?
   - *Did any of the children get tested for TB at home?*
   - *Did any of the children go to the clinic or hospital to be tested for TB?*
5. How important do you think it is for household members to be checked for HIV when someone is diagnosed with TB?
   - *Why is it important?*
6. Can you explain whether your family has had any checks for HIV since you were diagnosed with TB?
   - *If not, would you have liked your family to be checked for HIV?*
7. Where do you think these checks (for TB and HIV) should take place?
   - *Would it be better to happen at home, or at the clinic or hospital?*
8. Do you have any experience of having been visited at home by community care givers?
   - *If yes, can you tell me more?*
   - *What did the community care giver do during the visit?*
9. What would you feel about community care givers coming to the home to check people for TB and HIV?
   - *Would it be acceptable for a community care giver to visit the home for this?*
   - *What concerns would you have about a community care giver doing this?*
   - *Would you prefer for people focused on TB to visit your family at home?*
10. Other than checking the family for TB and HIV, is there anything else that you think it would be important for them to be checked for?
11. Are there any other health care services that should be provided for your family at this time?

***Any other comments***

Are there any final thoughts you have about TB household contact tracing?

***End of session***

Now we have come to the end of our discussion. Thank you for your participation. If you have any questions about your study participation, please contact us. Thank you.

#### Time ended (HHMM):

### S4

### INTERVIEW GUIDE: HOUSEHOLD CONTACTS

## OPTIMIZING THE EFFICIENCY OF HOUSEHOLD CONTACT TRACING FOR TB CONTROL IN SOUTH AFRICA

#### Introduction and Ground Rules

1. Obtain written informed consent first, before any data are collected.
2. Interviewer to introduce self. Thank you for taking the time to meet with us today. Our names are [*insert names*] _____________ and we would like to talk to you about TB contact tracing. We are doing this project to understand how we might improve TB contact tracing in the community, and particularly to see whether there are better ways to deliver contact tracing. We are interested in your views regardless of whether you have or have not got experience of TB contact tracing. We want you to be open when answering. There are no right or wrong answers in this discussion. Please feel free to tell us what you think.
3. Interviewer to explain the ground rules and terms of confidentiality for the interview:
   - The participant does not have to answer any question they do not want to.
   - The information you share will be handled in confidence. (in secret)
   - When we report back on the information collected in this discussion, your comments will not be able to be linked to you specifically.
   - We ask that you also agree not to share anything discussed in this room with others.
4. The discussion should take around one hour.
5. Interviewer to inform the interviewee that the in-depth interview will be tape recorded to make sure that all themes are captured. Turn the tape on and ask for verbal permission again to tape record, while the tape is running to verbally capture consent (this is a double check against the written consent). We will be recording the session because we don’t want to miss any of your comments. Although one of us may take some notes while we talk, we can’t write fast enough to get everything down on paper. As we are recording, please try to speak loudly so that we don’t miss your comments.

#### Themes to be explored

1. Knowledge and experience of TB (including TB contacting tracing and acceptability)
2. Knowledge and experience of HIV (including HIV counselling and testing and preference for HIV counselling)
3. Preference for any additional health services during a contact tracing visit

#### Time started (HHMM):

#### Questions

- 1. **Knowledge and experience of TB**
     - *Have you heard about a disease called TB (tuberculosis)? Can you explain to us the type of information on TB that you heard about?*
     - *Do you know how tuberculosis can be spread? If yes, tell me more.*
     - *Can you talk about TB in your house? If yes or no, what are some of the reasons?*
     - *Have any health workers visited you since someone in your house was diagnosed with TB? What did you like/dislike when your house was visited?*
     - *How could we make it better for you or other people in your community when health workers visit you?*
     - *What do you think your neighbours would say if they saw a health service vehicle at your house? Does this make you feel good or bad? Would it be better or worse if the vehicle didn’t have signs on it?*
  2. **HIV testing**
     - *Tell me what you know about HIV and about how it affects your community?*
     - *Describe your experience of being tested for HIV? (Probe on where and when HIV testing took place)*
     - *How do you feel about health workers offering you HIV testing in your house? Please tell why you would choose this as an option or not support this method of testing*
     - *What do you think about HIV testing being done at the same time as being checked for TB?*
     - *Did you test for HIV recently? Can you please describe to us what factors influenced your decision to test for HIV?*
     - *How did you feel about the process?*
     - *Were there any difficulties with HIV testing in your house?*
     - *Is there anything you think could be done differently when health workers visit your house?*
  3. **Preferences for contact tracing**
     - *Please describe your experience of TB contact tracing*
     - *Would you have preferred to go visit a clinic or have health workers visit you at home; what are some reasons for saying this?*
     - *What do you understand by the term ‘community care giver’?*
     - *Can you tell me about any experience you have of being visited by a community care giver?*
     - *What would you feel about a community care giver being the person that checks the family for TB and HIV?*
     - *When is the best time for us to visit you at your home to provide health services? During the day or in the afternoon after work?*
     - *When health staff visit you at home, are there any other things you would like them to help you with? Blood pressure or diabetes check?*
  4. Would you allow health workers to visit your household again in the future for household contact tracing of TB? Can you explain your answer?

***Any other comments***

Are there any final thoughts you have about TB household contact tracing?

***End of session***

Now we have come to the end of our discussion. Thank you for your participation. If you have any questions about your study participation, please contact us. Thank you.

#### Time ended (HHMM):
