# Supplementary material for: Exploring the promise and reality of ward-based primary healthcare outreach teams conducting TB household contact tracing in three districts of South Africa
Source: PLoS One. 2021 Aug 13;16(8):e0256033. doi: 10.1371/journal.pone.0256033 (PMC8362963; doi:10.1371/journal.pone.0256033)
Supplement: S2 File — (DOC) [file pone.0256033.s002.doc]

### FOCUS GROUP GUIDE: COMMUNITY STAKEHOLDERS

## OPTIMIZING THE EFFICIENCY OF HOUSEHOLD CONTACT TRACING FOR TB CONTROL IN SOUTH AFRICA

#### Introduction and Ground Rules

1. Obtain written informed consent first from all participants, before any data are collected.
2. Interviewer to introduce self. Thank you for taking the time to meet with us today. Our names are [*insert names*] __________ and we would like to talk to you about TB contact tracing. We are doing this project to understand how we might improve TB contact tracing in the community, and particularly to see whether there are better ways to deliver contact tracing. We are interested in your views regardless of your experience with TB contact tracing. We want you to be as open and honest when answering. There are no right or wrong answers in this discussion. Please feel free to tell us what you think, regardless of whether you agree or disagree with what you hear. We don’t expect everyone to share the same opinions. It is very important that we hear all your opinions.
3. Interviewer to explain the ground rules and terms of confidentiality for the focus group discussion:
   1. The participant does not have to answer any question they do not want to.
   2. The information you share will be handled in confidence. (in secret)
   3. When we report back on the information collected in this discussion, your comments will not be able to be linked to you specifically.
   4. We ask that you also agree not to share anything discussed in this room with others.
4. The discussion should take between one and two hours. We will have a break somewhere in the middle.
5. Interviewer to inform the participants that the focus group discussion will be tape recorded to make sure that all themes are captured. Turn the tape on and ask for verbal permission again to tape record, while the tape is running to verbally capture consent (this is a double check against the written consent). We will be recording the session because we don’t want to miss any of your comments. Although one of us may take some notes while we talk, we can’t write fast enough to get everything down on paper. As we are recording, please try to speak loudly so that we don’t miss your comments.

#### Themes to be explored

1. Motivators and barriers to diagnosis and treatment of household contacts
2. Internal or external factors that influence HHCT
3. Gaps in HHCT
4. Suggestions and plans for improvement/change

#### Time started (HHMM):

#### Questions

- 1. **General**
- *Please tell us a little about your role within the community*
- *Can you give us a brief description of the community that you represent?*
- *What types of health services are being provided in your community?*
- *In your opinion, what services do you think are important or needed in your community which is not currently available?*
  1. **Understanding of health services provided in the community**
- *Have you heard about a disease called TB (tuberculosis)? Can you explain to us the type of information on TB that you heard about?*
- *In your opinion, how do you think TB is affecting your community?*
- *Can you tell us about the health services offered in the community that helps people with TB and their families?*
- *Can you describe to us what people in the community do if they have TB?*
- *In your opinion, can you describe the reasons why people with TB may not access the services that are currently available to them?*
- *In your opinion, can you describe which services people with TB access most often and why?*
- *In your opinion, what health services do you think people with TB need that is not currently being provided?*
  1. **Experience and acceptability of TB contact tracing**
- *In this community, who provides TB services?*
- *Can you describe to us your understanding of TB contact tracing? (Use this probe only if participants do not know what TB contact tracing means: i.e. where health staff visit the houses of families in your community when a person has been diagnosed with TB to check if anyone else in the house has TB and to offer them a chance to test for HIV)*
- *How would you describe the activities that take place when health staff visit these households?*
- *How do you feel about health staff visiting homes of people in your community? Why do you feel that way?*
  1. **Perception of Community Care Givers (CCG’s)**
- *Can you describe your understanding of Community Care Givers (CCG’s) and their role within health services?*
- *Explain any general, positive or negative experiences that you had or know of regarding CCGs working in this community*
- *Please describe the community’s reaction to the work that CCGs do. What do you think are the thoughts and feelings from community members toward CCGS working in the community? (Probe on support or resistance from community)*
- *How would you feel about CCG’s providing health services in your community? What would be reasons for and against this?*
- *What do you think about CCGs being involved specifically with TB contact tracing? Please tell us about any concerns about this?*
- *How do you think the community would feel about sharing information with CCGs, especially if the CCG is someone that he or she knows from the community?*
- *Can you describe to us the motivators or challenges that community members could experience when talking to a CCG about their health status or other personal information?*
  1. **Other services that should be provided within the community**
- *What other health services would you want to see provided in your community?*
- *Do you feel more health services should be provided in your home, rather than at the clinics and hospital? Can you tell us what services these should be?*
- *Do you feel there is a need to speak more about HIV? Why do you feel that way?*
- *Please describe if you would prefer to test for HIV in your house as opposed to in the clinic or in the community? What would be the reasons for your choice*
- *How else do you think we can prevent TB from spreading in your community?*

***Any other comments***

Are there any final thoughts you have about TB household contact tracing?

***End of session***

Now we have come to the end of our discussion. Thank you for your active participation. If you have any questions about your study participation, please contact us. Thank you.

#### Time ended (HHMM):
